# Redefining the Immunobiology of Organ Transplantation for New Clinical Horizons

**DOI:** 10.1111/sji.70045

**Published:** 2025-07-27

**Authors:** Masoud H. Manjili

**Affiliations:** ^1^ Department of Microbiology and Immunology Virginia Commonwealth University (VCU) School of Medicine Richmond Virginia USA; ^2^ VCU Massey Comprehensive Cancer Center Richmond Virginia USA

## Abstract

Traditional organ transplantation relies on the Self–Non‐self (SNS) model of immunity, focusing on donor–recipient compatibility and aggressive immunosuppression to prevent acute rejection. Although effective early, this strategy does not prevent chronic rejection and cannot account for operational tolerance, failure of perfectly HLA‐matched grafts, or the occasional spontaneous acceptance of a fully mismatched organ. The adaptation model of immunity offers a different lens. In the thymus, “central adaptation” programs T cells to recognise self‐peptide–MHC (pMHC) so they can later recognise different tissues to facilitate tissue repair and homeostasis. Whether a graft thrives or fails depends on how quickly this self‐oriented circuitry can operate. Autografts and isografts arrive with their own extracellular‐matrix (ECM) “memory”, and recipient T cells immediately recognise their pMHC, triggering tissue‐remodelling responses. Allografts must adapt to new ECM—a transition that is associated higher levels of graft injury allowing indirect antigen presentation. Until adaptation is complete, recipient T cells mount cytotoxic rather than reparative responses because of antigen cross‐presentation, during which the graft relies on donor‐derived tissue‐resident memory T cells (T_RM_) to maintain integrity. Therapeutically, interventions that preserve or expand graft‐borne T_RM_, or that pharmacologically enhance adaptation‐receptor signalling, could hasten this donor‐to‐host reprogramming. By replacing blanket immunosuppression with targeted promotion of tissue‐remodelling immunity, the adaptation model charts a path toward long‐term graft survival without the lifelong risks of today's regimens.

## 1 Introduction

Organ transplantation represents a significant advancement in the treatment of organ failure, offering a crucial lifeline to numerous patients. Despite its transformative role, success in this field is compromised by several challenges, predominantly immune rejection. Historically, the Self–Non‐self (SNS) model of immunity has been the framework for understanding this rejection, characterised by the immune system identifying the transplanted allograft as alien and initiating a detrimental response. This model has led to the prevalent use of immunosuppressive treatments, which are designed to mitigate acute rejection by dampening the immune response. Although these treatments are initially effective, they are accompanied by severe drawbacks, such as heightened vulnerability to infections and cancers and notably, they do not avert chronic rejection, which is a primary cause of long‐term graft failure.

A range of compelling evidence now underscores the inadequacies of the SNS model in fully explaining transplant outcomes. For instance, certain patients achieve what is known as operational tolerance, where graft function is maintained with minimal or no use of immunosuppressives [1, 2]. On the other hand, cases of rejection can still occur [3], even with perfect HLA matching [3], and paradoxically, prolonged use of immunosuppressants can even promote chronic rejection [4]. Moreover, in instances of spontaneous kidney allograft acceptance, donor‐derived skin grafts are often also accepted, indicating that alloreactive immune responses could actually facilitate graft tolerance instead of solely triggering rejection [5]. These insights indicate that factors beyond simple alloreactivity are at play, revealing limitations in the SNS model to fully encapsulate the immunobiology of transplantation.

Originally derived from observations of immune responses to pathogens, the SNS model portrays the immune system as a defensive entity focused on eradicating external threats. This view, however, neglects the nuanced role of the immune system in tissue homeostasis. Immune cells not only act as agents of defence but are crucial in maintaining tissue integrity, aiding in repair, and regulating cellular balance. These processes are not governed by traditional tolerance mechanisms [6, 7] but rather through the active involvement of effector immune cells in homeostasis [8].

In transplantation, the goal should shift from broadly suppressing immune responses to engaging immune responses that support graft integration and ensure tissue stability. Current methods focus heavily on immune suppression and overlook the adaptive and regulatory abilities of the immune system, which are essential for long‐term graft survival. This essay presents the adaptation model of immunity, which recasts self‐reactive T cells as first‐responders for tissue repair, not agents of destruction.

## 2 Transplantation in the Pre‐Immunology Era

The realm of organ transplantation has evolved significantly over the past century, marked by pivotal breakthroughs in surgical techniques, immunology, and pharmacology. These advancements have shifted organ transplantation from a high‐risk, experimental procedure to a critical, life‐saving therapy for thousands of patients globally.

In 1902, French surgeon Alexis Carrel, while working in the United States, initiated pioneering organ transplantation experiments in animals. He devised innovative methods for vascular anastomosis, the surgical connection of blood vessels, which proved crucial for linking donor organs with the recipient's circulatory system. Carrel's groundbreaking techniques laid the groundwork for future advances in organ transplantation, ultimately earning him the Nobel Prize in Physiology or Medicine in 1912 [9]. In 1909, after approximately 100 experimental transplants, Ernst Unger successfully transplanted a kidney from a fox terrier into a boxer dog, with the kidney functioning and producing urine for 14 days [10]. In the 1930s, surgeon Yuri Voronoy ventured to perform the first human kidney transplants using deceased donors [11]. However, these early attempts were largely unsuccessful due to the then‐limited understanding of the immune response to foreign organs and the surgical challenges faced. A landmark success came in 1954 when Joseph Murray and John Merrill performed the first successful kidney transplant between identical twins at the Peter Bent Brigham Hospital in Boston [12]. This particular transplant circumvented the issue of immune rejection, as the twins were genetically identical, thus preventing any immune response against the transplanted organ. Murray's contributions to the field of organ transplantation were recognised with a Nobel Prize in 1990.

## 3 Immunobiology of Transplantation: The Traditional SNS Model

Transplant immunology has its foundations in the seminal work of Peter Medawar. His research in 1944 revealed that the immune system's response, particularly through lymphocyte infiltration, was pivotal in rejecting genetically dissimilar homografts, while autografts were accepted and healed seamlessly [13]. Medawar also noted that both dizygotic and monozygotic twins could accept grafts from each other but rejected those from unrelated third‐party donors [14]. Drawing inspiration from Macfarlane Burnet's theoretical concepts in immunology [15], Medawar proposed that the immune system could distinguish “self” from “non‐self”, Burnet's hypothesis was inspired by Ray Owen's work, which showed that twin cattle exhibited red blood cell chimerism, a rare phenomenon in twins of other placental species, including sheep and humans [16]. The initial finding about acquired tolerance was published in the 1953 Nature paper [17]. Later on, Billingham, Brent and Medawar reported that following the injection of late‐stage mouse embryos [18] or neonates [19] of an inbred strain with cell suspensions from another strain, the foreign grafts were accepted as “self”. Moreover, Avrion Mitchison, a former PhD student under Medawar, explored the characteristics of immunological memory in 1955 [20].

Medawar's contributions laid the groundwork for our current understanding of immune‐mediated graft rejection across species, earning him the Nobel Prize in 1960. According to this model, the immune system's primary function is to distinguish between “self” (the body's own tissues) and “non‐self” (foreign invaders such as pathogens). When a foreign substance, referred to as a non‐self‐antigen, enters the body, the immune system mounts an attack to eliminate the threat. When a donor organ is transplanted into a recipient, the recipient's immune system recognises the donor's cells as non‐self, primarily due to differences in major histocompatibility complex (MHC) molecules as well as minor histocompatibility antigens (mHA) presented to T cells in the groove of MHC [21]. This recognition triggers an alloreactive immune response, where T cells and B cells are activated to attack the transplanted organ, leading to graft rejection. To mitigate this immune response, transplantation medicine has relied heavily on immunosuppressive therapy. Donor–recipient compatibility, based on HLA matching, also plays a critical role in minimising the immune system's recognition of the graft as foreign. In 1962, the first kidney transplant between unrelated individuals was successfully performed using immunosuppressive therapy. In 1962, Thomas Starzl performed the world's first successful liver transplant in a human, marking a major advancement in transplant surgery [22]. Starzl would later refine immunosuppressive therapy for liver transplantation and become one of the most prominent figures in transplant surgery. In 1967, Christiaan Barnard performed the first successful human heart transplant in Cape Town, South Africa, using a heart from a deceased donor [23]. Although the patient survived for only 18 days, this groundbreaking surgery demonstrated the potential for heart transplantation. Barnard's work spurred further innovations in heart transplantation techniques and immunosuppressive protocols. In 1979, the first successful human trials of cyclosporine in kidney transplantation were conducted by Roy Calne [24]. The drug significantly improved outcomes and became the cornerstone of immunosuppressive therapy for various organ transplants. During the 1980s, heart‐lung transplants became feasible due to improved surgical techniques and immunosuppression. The first successful heart‐lung transplant was performed by Bruce Reitz at Stanford University [25]. In 2000s, organ preservation techniques improved significantly, extending the time organs could be kept viable outside the body. The use of cold storage solutions and machine perfusion allowed more organs to be transported over longer distances, increasing the availability of organs for transplantation. In 2005, the first partial face transplant was performed in France on a woman who had suffered severe facial trauma [26]. This groundbreaking procedure represented a new frontier in reconstructive transplantation, combining surgical expertise with knowledge of immunosuppression. In 2014, the first successful uterus transplant was performed in Sweden, leading to the first live birth from a transplanted uterus [27]. This represented a breakthrough in reproductive medicine, offering new hope to women with uterine infertility.

While immunosuppression has been effective in minimising acute rejection, its significant drawbacks cannot be overlooked. Long‐term use of immunosuppressive medications heightens the risk of severe health issues including cardiovascular diseases, metabolic syndrome, bone loss, as well as opportunistic and community‐acquired infections. Moreover, these drugs contribute to an increased risk of malignancies due to the suppression of the overall immune system. Additionally, immunosuppressive drugs often fail to prevent chronic rejection, where the transplant gradually deteriorates over time due to fibrosis, vascular damage, and chronic inflammation. The traditional view of the immune system by the SNS model primarily regards it as a defence mechanism against foreign invaders, thus neglecting its comprehensive roles in self‐reactivity for maintaining tissue homeostasis and repair, as well as in supporting the functions of target organs.

## 4 Unresolved Challenges of the SNS Model in Organ Transplantation

Organ transplantation has advanced significantly, incorporating vital organs such as kidneys, livers, hearts, and lungs. Despite these advancements, our comprehension is still limited by traditional immune models, particularly the SNS model. While the SNS model has substantially influenced immunology and transplantation medicine, it provides a limited and simplified perspective on the immune system's role in establishing graft tolerance and maintaining tissue equilibrium. Although the phenomena of autograft acceptance and allograft rejection seem to validate this model, emerging evidence suggests that the mechanism driving rejection extends beyond mere alloreactivity, indicating that organ adaptation processes might play a pivotal role in the survival of the graft. This growing array of contradicting evidence against the SNS framework compels us to reassess existing paradigms and consider alternative models. These alternatives could potentially offer a more nuanced understanding of immune functions in transplantation, as further explored in the discussions that follow.

### 4.1 Less Matched Living Donor Organs Do Better Than Matched Cadaver Donor Organs After Transplantation

Clinical observations show that organs from less closely matched living donors often perform better than well‐matched organs from deceased donors following transplantation. This phenomenon is partially explained by the danger model, which posits that cadaveric organs sustain more damage. Such damage leads to an increased release of Damage‐Associated Molecular Patterns (DAMPs), further stimulating the maturation of antigen‐presenting cells (APCs) and the emission of co‐stimulatory signals vital for triggering T cell activation against the graft. This results in a stronger immune response and a higher chance of organ rejection [28, 29, 30]. In contrast, living donor organs experience less tissue damage, thus emitting fewer DAMPs and alarm signals, which enhances their capacity to be tolerated by the recipient's body. This variance highlights the importance of minimising graft damage to improve the chances of successful engraftment. For example, using antioxidants like superoxide dismutase (SOD) has been noted to alleviate oxidative stress in grafts, improving transplant outcomes, as demonstrated by Walter Land in a clinical trial with 44 kidney transplant patients [31]. Moreover, blocking co‐stimulatory signals (signal II) using anti‐CD40L in a study with six monkeys resulted in successful kidney transplants, where signal I alone was sufficient to induce tolerance without rejection [32]. Further emphasising the significance of co‐stimulatory signals, experiments with minor antigen HY–mismatched allografts showed that without MyD88 signalling in APCs, crucial for providing signal II, rejection did not occur [33]. Additional support comes from a study on germ‐free mice, where male skin grafts lasted significantly longer in germ‐free female recipients compared to specific pathogen‐free (SPF) mice, attributed to the reduced microbe‐induced DAMPs in the germ‐free environment [34, 35]. Interestingly, the danger model also sheds light on the effects of cyclosporin A (CsA), an immunosuppressant. CsA disrupts signal I (TcR signalling) in the T cells of the recipient, thwarting the establishment of tolerance [28]. This was illustrated in a study where liver transplants in rats induced tolerance, allowing acceptance of subsequent skin grafts from the same donor. However, administering CsA concurrently with the liver transplant prevented this tolerance induction, indicating that interfering with signal I impairs natural tolerising mechanisms [24].

While the danger model effectively explains the role of DAMPs and co‐stimulatory signals in graft rejection, it doesn't fully account for why organ transplants between identical twins typically yield better outcomes than allografts, despite similar donor conditions and surgical procedures. This limitation, shared with the SNS Model, shows that while these models describe mechanisms that trigger immune responses, they do not fully predict outcomes in graft acceptance or rejection. This underscores the need for more research into immune regulation to foster tolerance and enhance success in transplantation.

### 4.2 Allograft Survival in the Presence of Anti‐Graft Immune Response

Despite the well‐established link between anti‐donor antibodies and chronic graft rejection [36], some clinical and experimental studies have called this relationship into question, suggesting that tolerance can still be achieved despite an active anti‐graft immune response. Notably, patients who managed to sustain transplanted organs, some of them (2 out of 9 patients tested) showed anti‐donor class II antibodies [37]. Moreover, cases of prolonged graft survival in liver transplant recipients with significant HLA discrepancies further underscore the complex nature of immune tolerance in transplantation settings [1, 38]. This phenomenon suggests that graft survival does not solely hinge on the absence of immune reactions; rather, it may be supported by regulatory mechanisms that enable the immune system to coexist with the graft without leading to its rejection.

### 4.3 Persistence of Microchimerism Following Organ Transplant

Microchimerism is the presence of a small proportion of donor immune cells—less than 0.1% of total cells—from passenger haematopoietic cells in the recipient following an organ transplant. This phenomenon plays a critical role in the immunology of organ transplantation. Known also as mixed chimerism, this low‐level persistence of donor cells has been connected with graft tolerance in certain cases. However, the existence and functional significance of microchimerism presents a challenge to the traditional SNS model of immunity, which struggles to fully account for the occurrence of tolerance in the presence of foreign donor cells without ongoing immunosuppressive treatment. A natural occurrence of microchimerism can be observed during pregnancy when maternal immune cells become sensitised to paternal antigens from the child, and the child's immune system similarly becomes exposed to and recognises non‐inherited maternal antigens. This mutual sensitisation offers a model to understand potential interactions between persistent donor cells and the recipient's immune system post‐transplant. Clinical studies, including those by Starzl et al., have indicated that microchimerism may be linked with graft acceptance, notably in kidney, liver, or small bowel transplants [39]. However, microchimerism does not universally lead to successful transplantation outcomes. For instance, several clinical studies have shown no significant correlation between the persistence of microchimerism and graft acceptance in cadaveric kidney [40], liver [41] and heart [42] transplants. These mixed findings suggest that while microchimerism can contribute to immune tolerance under some conditions, it is not the definitive factor governing graft success.

The variable nature of these outcomes highlights the complexity of transplant immunobiology and suggests that factors such as the immune environment, tissue damage, and other regulatory mechanisms also play critical roles. Further investigation is essential to fully understand the precise impact of microchimerism on long‐term graft survival and to explore how this phenomenon could potentially improve transplant success rates without perpetual reliance on immunosuppression.

### 4.4 Operational Tolerance and Spontaneous Allograft Acceptance

A subset of transplant recipients demonstrates a remarkable capacity to maintain graft function with minimal or negligible immunosuppression, a phenomenon known as operational tolerance [1, 2]. This condition is typically seen in patients who have received allogeneic kidney or liver transplants and manifests in two main scenarios: (i) spontaneous tolerance arising from non‐adherence to immunosuppressive regimes, and (ii) deliberate withdrawal of immunosuppressants under clinical oversight [43]. Interestingly, operational tolerance appears to be more prevalent among paediatric patients, where it represents up to 60% of cases [1], in contrast to 20% in adults [44]. This suggests potential age‐related differences in immune adaptability or tolerance mechanisms.

Preclinical research has reflected these clinical observations, where spontaneous acceptance of both mouse kidney and rat liver allografts provides promising insights into the potential for long‐term graft survival without lifelong immunosuppression. Such findings carry substantial promise for transforming transplant medicine by reducing the adverse impacts of chronic immunosuppression. Despite the profound implications of these findings, the early immune processes critical for spontaneous graft acceptance, especially in mouse kidney transplants, are not yet well understood. Investigating these mechanisms may reveal new therapeutic targets to induce drug‐free tolerance in transplant recipients. Notably, spontaneous acceptance extends beyond kidney allografts to include donor‐derived skin allografts in preclinical models, suggesting that this tolerance is likely mediated by active immune regulation rather than mere immune ignorance [5, 45]. However, the proposed role of Tregs in fostering operational tolerance has not found substantial biological support in these instances, hinting at other immune‐regulatory pathways. Understanding these processes can pave the way for long‐term allograft acceptance without continuous immunosuppression, potentially revolutionising transplant outcomes and enhancing both graft survival and patient quality of life. The prospect of drug‐free transplantation marks a paradigm shift in the field with implications for both clinical practice and long‐term management of transplant recipients.

### 4.5 Allograft Acceptance Without Immunosuppressive Therapy

Emerging evidence, both from preclinical models and clinical studies, questions the necessity of lifelong immunosuppression for allograft survival. In murine models of heart and kidney transplantation, researchers have successfully achieved long‐term graft acceptance without immunosuppression, offering vital insights into the immune tolerance mechanisms relevant to transplantation [46]. These discoveries have profound implications for transplant medicine, suggesting that the immune system can, under certain conditions, be modulated to accept allogeneic tissue without continuous suppression.

In human clinical settings, a similar phenomenon has been observed. Notably, patients undergoing a combined kidney and bone marrow transplant have achieved stable graft survival with only temporary chimerism, where donor cells coexist with recipient cells briefly [47]. This indicates that even short‐term immune modulation involving donor‐derived haematopoietic cells may induce sustained graft acceptance. Further supporting this, studies involving humanised CD34^+^ NSG mice, reconstituted with human immune cells, have demonstrated that human T cells can tolerate HLA‐mismatched tumour cells, revealing a capacity for immune tolerance even amidst genetic differences [48]. This model underscores the possibility that particular immune‐regulatory mechanisms, potentially independent of HLA compatibility, can facilitate tolerance and prevent graft rejection. These collective findings highlight the adaptability of the immune system and its potential for achieving tolerance without long‐term immunosuppression. Unravelling the precise mechanisms behind this phenomenon could transform transplantation therapy, reducing reliance on immunosuppressive drugs and minimising its associated risks such as infections, malignancies, and chronic rejection. This shift could revolutionise the management of organ transplant recipients, presenting safer and more sustainable treatment outcomes.

### 4.6 Chronic Graft Rejection and Fibrosis

Chronic graft rejection, marked by fibrosis and vascular damage, is a leading cause of long‐term graft failure. It is increasingly acknowledged that this condition is spurred not only by immune responses but also by non‐immune factors. These non‐immune factors encompass tissue remodelling, fibrosis, and vascular alterations that gradually manifest within the graft. Significantly, the prolonged use of immunosuppressive medications can paradoxically fuel the development of chronic rejection [4]. For example, calcineurin inhibitors like cyclosporine, which are frequently administered to prevent acute rejection, are notorious for their nephrotoxic effects. These medications cause vascular damage, disrupt kidney function, and encourage fibrosis, thus contributing to a gradual decline in graft function. This nephrotoxicity presents a critical challenge in transplantation: Although essential for managing acute rejection, long‐term use of immunosuppressive drugs may initiate the very complications leading to chronic graft failure. These observations indicate that chronic rejection is influenced not only by immune alloreactivity but also significantly by non‐immune tissue damage and the adverse effects of extended immunosuppressive treatment. The role of fibrosis and vascular remodelling in chronic rejection highlights the urgent need for more comprehensive understanding of these non‐immune mechanisms.

### 4.7 Graft Versus Host Disease (GVHD)

The SNS model attribute GVHD to donor alloreactive T cells after allogeneic stem cell transplantation; however, evidence points to host‐derived, self‐reactive T_RM_ cells—re‐programmed from a homeostatic to an inflammatory state—rather than newly arrived alloreactive T cells, as key drivers of tissue damage [49, 50]. According to the adaptation model, that pre‐transplant conditioning and GVHD prophylactic regimens disrupt signal IV, especially within highly proliferative barrier tissues (skin, gastrointestinal tract, liver). Loss of this homeostatic axis converts T_RM_ cells from reparative managers into cytotoxic agents, accounting for the characteristic organ tropism and severity of GVHD [51]. Importantly, GVHD has been reported even following autologous stem cell transplantation [52, 53].

## 5 Immunobiology of Organ Transplantation

In the domain of special relativity, the observation of events significantly depends on the perspective of the observer. Analogously, in immunology, while empirical data forms the basis of scientific insights, the interpretations are influenced by the immunologists' adopted theoretical frameworks. Historically, the SNS model has been predominant for over 70 years, directing experimental designs, data interpretation, and the development of treatment strategies. This model has largely defined conventional views on central tolerance, peripheral tolerance, and the destructive nature of immune responses, often simplifying the intricate regulatory functions of the immune system in preserving tissue homeostasis and structural integrity [7, 54]. A thorough reevaluation of existing data uncovers the varied roles of the immune system in supporting tissue integrity non‐destructively.

**FIGURE 1:**
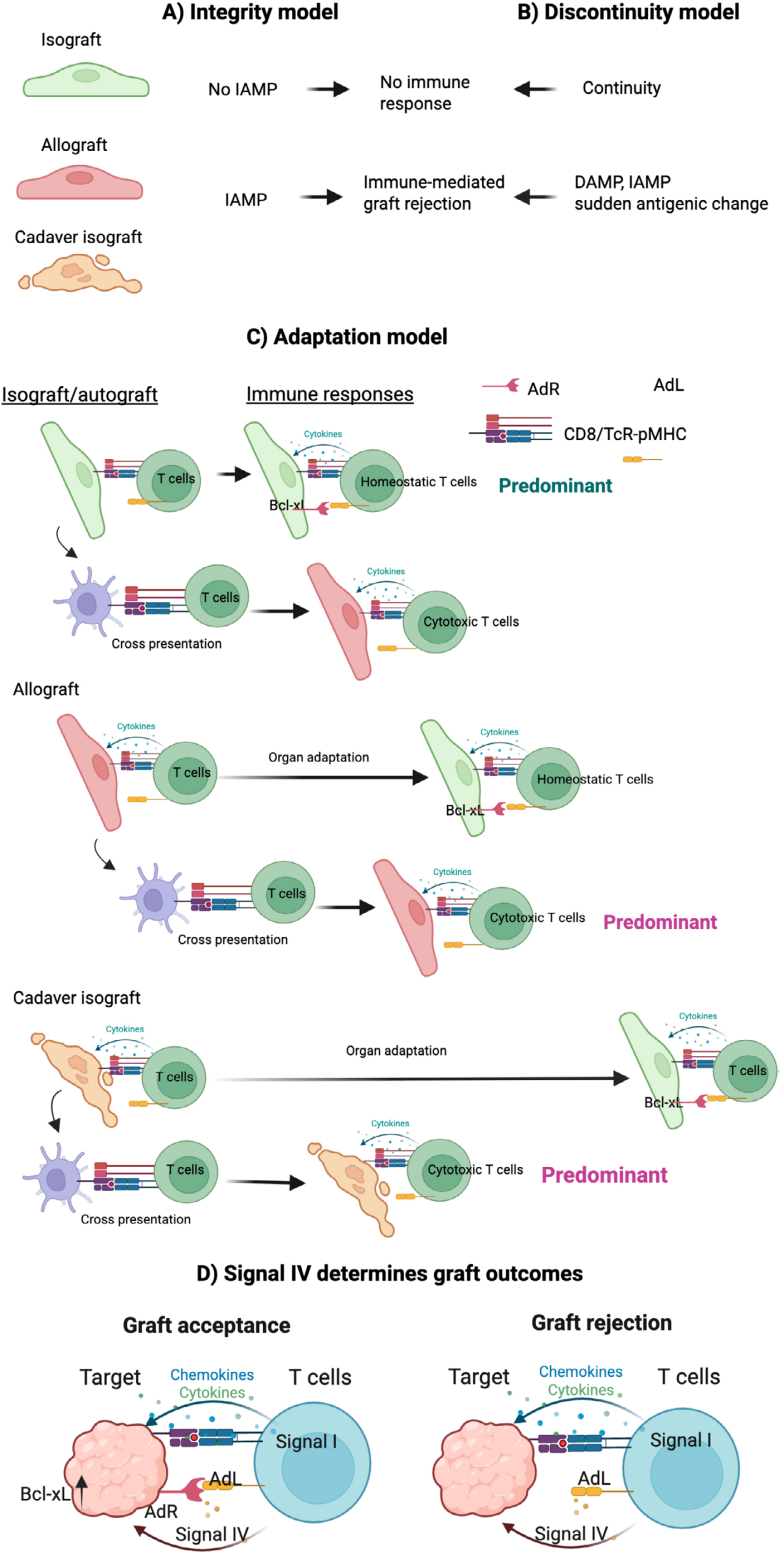
Three alternative/complementary models to the SNS and danger models. (A) A scenario extrapolated from the integrity model, explains rejection or acceptance of a graft depending of the presence or absence of IAMP on the graft for inducing immune‐mediated graft rejection. (B) According to the discontinuity model, any discontinuity in the form of DAMP or IAMP or sudden exposure to non‐self‐antigens could induce tissue‐rejecting immune responses. (C) According to the adaptation model, homeostatic immune responses is directly induced by the graft and become predominant because of less damaged graft, regardless of its antigenic source being allograft or isograft. Cytotoxic T cells are also induced by antigenic cross‐presentation to clear injured cells without harming healthy cells that express AdRs. Acceptance or rejection of the graft will be determined by the ability of the graft to re‐express AdRs, which could be faster for a living donor isograft than a living donor allograft, but cadaver donor graft takes longer to restore AdRs due to the graft carrying more damage. When allograft or cadaver graft adaptation is delayed, donor antigens are processed and presented indirectly by recipient APCs, skewing the immune response toward predominant cytotoxic rather than homeostatic pathways. (D) Graft acceptance is when signal IV (AdR‐AdL) is present during inflammatory immune response, whereas graft rejection occurs in the absence of signal IV or AdR loss on the graft. T cells express AdL in the form of secretory cytokines/chemokines or as a membrane receptor.

Zlatko Dembic's integrity model underscores the immune system's role in maintaining tissue integrity through recognising and reacting to integrity‐associated molecular patterns (IAMPs) or Signal III. These are molecular structures linked to compromised or altered tissue integrity [7]. Here, disrupted integrity triggers an immune response, whereas preserved integrity fosters tolerance. It could be extrapolated from the integrity model that graft rejection could be due to the expression of IAMPs in altered graft (Figure 1A). The discontinuity model by Thomas Pradeu proposes that the immune system responds to abrupt changes in the organism such as dangers, non‐self‐antigens, loss of tissue integrity, and structural changes, perceived as discontinuity [55]. According to this model, ongoing exposure to self‐antigens or minor disturbances generally leads to tolerance or anergy, while dramatic alterations, like the emergence of pathogens, tumours, or sharp metabolic shifts, invoke immune responses by recognising these disrupted or missing normal patterns [55]. Accordingly, graft rejection could be because of sudden antigenic change in the allograft but not isograft seen by the recipient's immune system (Figure 1B). Contrasting the traditional focus on destruction in the SNS model, the adaptation model presents an enriched perspective of immune cell‐target cell communication through Signal IV. This model asserts that all somatic cells express adaptation receptors (AdRs) connected to anti‐apoptotic pathways, which include members of the Bcl‐2 family (e.g., Bcl‐xL, Mcl‐1) and serpins (e.g., SPI6/PI9), intercepting granzymes [56], alongside survival pathways such as PI3K/Akt. These AdRs engage with Adaptation Ligands (AdLs) or co‐receptors on immune cells, facilitating pro‐survival signals to shield target tissue from damage during self‐reactive, homeostatic immune responses [8].

According to the adaptation model, the primary role of self‐reactive immune responses is to participate in tissue homeostasis and function when no infection or tissue disruption is present [8]. To this end, RAG‐deficient mice exhibit impaired Notch signalling in colonocytes and defective epithelial differentiation—defects that can be reversed by adoptive transfer of T cells [57]. They also display reduced angiogenesis and delayed wound healing [58], along with compromised arteriogenesis [59]. Under this model, mHAs are neither inherently “self” nor “foreign” at the scale of individual amino acids. The TcR's CDR3 loop interacts directly with the peptide side chains presented by MHC, regardless of whether those residues derive from donor or host proteins [60]. Autografts and isografts arrive with intact tissue “memory” cues, allowing host self‐reactive T cells to trigger a predominantly homeostatic repair response immediately. This rapid interaction promotes swift re‐expression of AdRs and speeds graft integration. Any cells damaged during procurement or implantation are dealt with through antigen cross‐presentation, which confines cytotoxicity to the injured cells while sparing the surrounding healthy, AdR‐expressing tissue (Figure 1C). Numerous studies have demonstrated both the presence and the plasticity of tissue memory. For example, fibroblasts explanted from different body sites retain location‐specific DNA‐methylation and Hox‐gene signatures through many passages, guiding region‐appropriate ECM and cytokine profiles [61]. In skeletal muscle, resistance training leaves hypomethylated enhancers at growth‐regulating genes; months later, these loci are re‐activated more rapidly because of established memory, explaining faster hypertrophy on retraining [62]. Liver regeneration memory manifested as a chromatin state that primes immediate‐early genes facilitates accelerates regeneration upon a second injury triggers [63]. Such memory and its adaptability of the liver in producing bile are also evident after gallbladder removal. Allografts, however, must first remodel to adapt the recipient's ECM architecture. During this vulnerable period of graft injury, donor antigens are captured and cross‐presented by host APCs, eliciting predominant cytotoxic T‐cell responses (Figure 1C). Our group and others have shown that hepatocytes and sinusoidal endothelial cells can directly activate T cells in situ—a pathway that predominates in liver‐specific, tissue‐remodelling responses [64, 65, 66, 67, 68]. By contrast, indirect (cross‐) presentation of antigen by APCs, as occurs after vaccination, preferentially drives cytotoxic effector T cell responses. In cadaver donor isografts, AdRs expression is disrupted and its re‐emergence delayed, reflecting the greater ischemic and inflammatory injury the organ sustains before implantation and promote antigen cross‐presentation (Figure 1C). It was demonstrated that similar‐sized patellar tendon allografts manifest a slower rate of biologic incorporation compared to autografts [69, 70]. Such adaptation of allografts may be achieved by continuous infusion of growth factors [71]. Different rates of graft adaptation can also be influenced by somatic variations. Research indicates that humans exhibit significant genetic variation in somatic tissues, which adapt to their host [72]. Organs typically adjust to specific conditions such as collagen cross‐linking, stiffness, the rate of ECM remodelling, and metabolic conditions inherent to their original environment. Patterns of integrin expression also differ among individuals, shaped by both genetic factors and the tissue's environment. For instance, some individuals naturally express higher levels of integrins like α5β1 or αVβ3, which influences their responses in fibronectin‐rich environments. These variations in integrin interactions with ECM components play crucial roles in cellular processes such as adhesion and differentiation. Recent studies have shown that memory formation extends beyond immune cells. Tissues such as the kidneys and nerve cells have been found to store memory, responding to repeated stimuli in a manner similar to brain cells [73]. The memory responses of these organs are influenced by both the number of training pulses and their precise temporal spacing [73]. As a result, these differences might explain why some grafts exhibit quicker or more effective tissue repair responses because of spatial memory, thus enhancing their AdRs compared to others. The critical factor is not immune alloreactivity, but the graft's ability to adapt to the host microenvironment that is genetically distinct from its tissue of origin.

Alongside tissue‐remodelling immune responses, cytotoxic responses are also triggered after organ transplantation. In this case, if the target tissue loses its AdRs, it becomes susceptible to cytotoxic cytokines/chemokines released from T cells and fails to receive signal IV, leading to disrupted tissue homeostasis (Figure 1D). AdLs can be membrane‐bound receptors like PD‐1, which binds to the bidirectional receptor PD‐L1/B7‐H1 to convey survival signals to target cells [74, 75, 76, 77, 78]. Alternatively, they might be cytokines released by T cells, such as soluble TNF‐α that binds to TNFR1 inducing apoptosis, or membrane‐bound TNF‐α that binds to TNFR2 promoting survival [79, 80]. The regulatory role of immune responses in maintaining homeostasis is highlighted in healthy individuals. For instance, activated Th17 cells control the mucosal barrier function in the gastrointestinal tract without causing damage [81], and IFN‐γ‐producing meningeal T cells regulate excessive neural excitability and social behaviour in the CNS, again without deleterious effects [82]. Recent findings reveal that T cells traffic into the healthy brain—even in the absence of infection or disease—to support normal brain function by secreting IFN‐γ, thereby maintaining CNS homeostasis through the fat–brain and gut–brain axes [83]. In the gut, IFN‐γ regulates intestinal epithelial homeostasis by regulation of β‐catenin signalling pathways [84]. Also, IFN‐γ–producing Th1 cells support mammary tissue homeostasis by restraining luminal differentiation and limiting ductal branching [85]. These instances emphasise the inflammatory immune response's supportive role in sustaining tissue health rather than merely causing destruction. They could also destroy damaged or injured cells that had lost AdRs in order to facilitate homeostasis.

### 5.1 Central Adaptation Generates Self‐Reactive T Cell for Participating in Tissue Homeostasis

The central adaptation model introduces a paradigm shift in understanding T cell selection within the thymus, challenging conventional immunological theories that primarily emphasise central tolerance through the elimination of self‐reactive T cells to prevent autoimmunity. It is important to note that central adaptation builds on the same empirical observations that underpin central tolerance, yet—as outlined below—it offers a more comprehensive interpretation of those findings. According to the traditional SNS model, T cells undergo cortical positive selection to ensure self‐reactive MHC restriction, followed by medullary negative selection to eliminate high‐affinity self‐reactive T cells, thus preventing autoimmunity. However, this model overlooks the essential roles self‐reactive T cells play in sustaining tissue health. In the central adaptation model, T cells experience a dual‐layered positive selection process. Initially, cortical positive selection confirms self‐reactive MHC restriction and eliminates T cells that either fail to recognise self‐peptide–MHC (pMHC) complexes or lack a crucial signal I from the pMHC‐TcR interaction. Subsequently, during medullary positive selection, only those functional T cells that can activate the anti‐apoptotic Bcl‐xL pathway through CD28 co‐stimulation are retained—a conclusion supported indirectly by the well‐documented up‐regulation of Bcl‐xL downstream of CD28 signalling during T‐cell activation [86, 87]. Conversely, T cells that are unable to activate anti‐apoptotic pathways upon receiving the co‐stimulatory signal II are eliminated. Moreover, functional T cells that efficiently receive survival signals during co‐stimulation are positively selected [8, 88]. This dual selection process ensures the survival of functional T cells adept at handling both signal I and signal II. These T cells, expressing AdRs, are equipped to receive pro‐survival signals during activation and contribute to the immune system's homeostatic response (Table 1). Conversely, T cells that escape medullary positive selection are more susceptible to apoptosis upon activation. In contrast to the conventional negative‐selection model, the central adaptation model accounts for how thymic atrophy—whether induced by sepsis or ageing—alters thymic selection and reshapes the T‐cell repertoire. Sepsis is characterised by significant lymphopenia [89, 90] associated with thymic atrophy [91] where autoimmunity development is diminished [92], potentially due to the presence of aberrant T cells that bypassed positive selection in the medulla. These defective T cells display notably lower levels of Bcl‐xL, rendering them more prone to apoptosis [93]. Similarly, in malnourished children experiencing thymic atrophy, lymphopenia occurs due to lymphocyte apoptosis rather than autoimmunity [94, 95]. Also, aging is associated with a decline in naïve T cells [96], reduced TcR repertoire diversity [97], and diminished effectiveness in priming [98]. Empirical data contradict the belief that medullary negative selection completely eradicates high‐affinity T cells. Research involving *Aire*‐deficient mice demonstrates parallel TcR Vβ repertories when compared to wild‐type mice, with no substantial signs of autoimmunity, aside from mild autoimmune‐like symptoms such as dry eyes [99]. Additionally, in transgenic mice where specific antigens are presented in medullary thymic epithelial cells under *Aire* regulation, the elimination of corresponding antigen‐specific CD4^+^ T cells does not occur [100]. Similarly, high‐affinity H‐Y TcR transgenic CD8^+^ T cells continued to exist in male mice without leading to autoimmunity [101]. After all, medullary T cells that express a high‐affinity TcR for self‐antigens are not eliminated but instead become CD4^+^ Tregs [102, 103, 104] or CD8^αα+^ Tregs [105, 106, 107]. Recent work by Bilgic‐Eltan and colleagues [108] reveals pronounced T cell lymphopenia and a notable drop in naive T cells following thymectomy in infants with congenital heart disease. Intriguingly, although three patients developed autoantibodies, none displayed clinical autoimmunity. These findings echo previous data indicating a progressive, long‐lasting decline in cellular immunity post‐thymectomy [109], challenging the long‐held assumption that thymic disruption should unleash autoreactive T cells and trigger autoimmunity.

**TABLE 1:** Central tolerance vs. central adaptation.

	Central tolerance	Central adaptation
Cortex	Positive selection of self‐reactive T cells Maturation of double positive T cells into single positive T cells	Positive selection of self‐reactive T cells that survive signal I Maturation of double positive T cells into single positive T cells
Medulla	Negative selection: elimination of self‐reactive, high‐affinity T cells and survival of self‐reactive, low‐affinity T cells	Positive selection of self‐reactive T cells that survive signal II, and elimination of defective T cells that commit suicide upon receiving signal II

These findings challenge the conventional view of thymic negative selection, suggesting instead that what has been interpreted as “negative selection” reflects the removal of inherently defective T cells. Meanwhile, functionally competent T cells undergo positive selection in both the cortical and medullary regions of the thymus. Recent research has begun to move away from the rigid concept of medullary negative selection, proposing that central tolerance mainly prunes but does not wholly eliminate autoreactive T cells [110]. Support for this notion comes from findings showing that CD8^+^ T cells specific to both self and non‐self‐antigens occur at comparable frequencies in healthy individuals, without triggering autoimmunity [111]. Under the revised model, self‐reactivity is not an aberration but a defining feature of every mature T cell. This intrinsic specificity allows T cells to patrol host tissues, secrete remodelling chemokines and growth factors that guide repair, and apply tightly regulated cytotoxicity to cull injured or senescent cells together—supporting day‐to‐day tissue homeostasis. In a murine model tracking the progression of metabolic‐associated fatty liver disease (MAFLD) to hepatocellular carcinoma (HCC), single‐nuclei RNA‐seq analyses showed that more than 75% of intra‐hepatic immune cells were dedicated to homeostatic functions—secreting tissue remodelling chemokines—whereas only ~5% displayed a cytotoxic phenotype. Notably, these cytotoxic cells targeted activated fibroblasts rather than tumour cells, promoting fibroblast turnover and thereby restoring a liver microenvironment that is inhospitable to tumour growth instead of directly attacking the carcinoma [65]. In this framework, there is no “tolerance” in the classical sense; rather, self‐reactive T cells are stimulated by self‐tissues to aid in tissue repair, homeostasis, and the preservation of tissue integrity. This model indicates a paradigm shift in immunology, emphasising the adaptive and regulatory roles of T cells in sustaining tissue health rather than merely preventing autoimmunity. For instance, it is estimated that around 150,000 T cells are present in the cerebrospinal fluid (CSF) of healthy individuals, where they work collaboratively with resident immune cells of the central nervous system (CNS) to regulate its normal physiological functions [112]. Supporting this idea, studies have demonstrated that mice engineered to express T cells that recognise endogenous myelin basic protein (MBP) show enhanced hippocampal neurogenesis and improved spatial learning compared to counterparts lacking such T cells [113]. Moreover, MHC II^−/−^ mice, which lack CD4^+^ T cell responses, display impaired performance in the Morris water maze (MWM), a test for assessing spatial learning and memory [114]. One explanation for this observation is that this cognitive impairment results from the inability of CD4^+^ T cells to recognise and interact with MHC class II‐positive astrocytes, which play a crucial role in learning and memory [115]. Additionally, astrocytes promote the differentiation of naïve CD4^+^ T cells into Th1 cells [116]; these IFN‐γ–producing Th1 cells then re‐engage astrocytes through the damage‐limiting “signal IV” pathway, preserving tissue integrity during immune activation [117]. Furthermore, even in tissues that lack MHC class II expression, CD4^+^ T cells can indirectly contribute to tissue homeostasis by aiding CD8^+^ T cells in targeting MHC class I‐positive cells, showcasing the immune system's diverse roles in preserving tissue health. In nude mice, which are deficient in functional T cells, lower levels of brain‐derived neurotrophic factor (BDNF) and cognitive deficits, including poor performance in the MWM task, have been observed. Remarkably, these deficits can be reversed by adoptively transferring T cells from wild‐type mice [118]. Activated T cells in humans are known to release bioactive BDNF, essential for neuronal survival and cognitive function [119], further underscoring the vital role of the immune system in not only defending against threats but also in supporting and enhancing neurological functions. In the brain, CD8^+^ T_RM_ cells have been demonstrated to produce IFN‐γ, thereby offering protection to the brain against infections without causing damage to the nervous system [120]. In humans, T_RM_ cells demonstrate tissue remodelling abilities by producing the epidermal growth factor receptor (EGFR)‐ligand amphiregulin (AREG), which promotes epithelial cell regeneration. Notably, blocking EGFR signalling or the cytokines IFN‐γ and TNF has been shown to inhibit this tissue remodelling process [121]. In mice, IFN‐γ produced by tissue‐resident NKT cells has been reported to regulate intestinal epithelial cell homeostasis, with the ablation of NKT cells or CD1d in vivo leading to altered epithelial cell proliferation [122]. CCL2 has been identified as a factor that heightens excitability in glutamatergic neurons during synaptic transmission, emphasising the critical role of the immune system in normal neuronal operations [123]. Additionally, human activated T cells exhibit neuroprotective functions, underscoring the significance of immune cells in safeguarding the brain from degeneration [124]. Recent studies have highlighted both neuroprotective and neurodegenerative roles of inflammatory T cells and NK cells in experimental models of trauma, illustrating the complex functions of immune cells within the CNS [125]. Such homeostatic function of effector T cells has been viewed as another specialised T cell population as Tx cells, a hypothetical subset of self‐reactive T cells that perform interoceptive homeostatic functions in various tissues [126]. Nevertheless, conventional negative selection in the thymus cannot explain the presence of harmless self‐reactive T cells.

### 5.2 Peripheral Adaptation Is the Process Through Which Transplanted Organs Manage to Tolerate Ongoing Homeostatic Immune Responses

Traditionally, peripheral tolerance is understood as the suppression of self‐reactive T cells by regulatory immune cells such as Tregs, Bregs, Mregs, MDSCs, and tolerogenic DCs. However, a different perspective provided by the adaptation model suggests that these regulatory cells might not be tolerogenic in the traditional sense. Instead, they are activated to produce a unique cytokine profile distinct from that of cytotoxic T cells. A critical distinction in this model is its emphasis on the target tissues, which express AdRs that enable them to tolerate both inflammatory and regulatory immune responses. These immune responses have AdLs or co‐receptors that interact with the tissue AdRs, promoting tissue protection and adaptation rather than destruction. Where the SNS paradigm frames rejection as alloreactivity that must be quelled through global immunosuppression or tolerance induction, the adaptation model views graft outcome as the balance between tissue‐remodelling immunity and cytotoxic immunity as well as adaptability of the graft in re‐expression of AdRs (Table 2). RAG‐deficient female mice accepted both a female and a male RAG‐deficient skin graft. When the recipients were later reconstituted with normal female fetal‐liver cells, the male graft was rejected whereas the female graft remained intact [127]. The original authors argued that recognition of a single male‐specific H‐Y antigen was sufficient to initiate rejection. However, in a parallel experiment they reconstituted similarly engrafted recipients with a RAG‐deficient thymus—incapable of TcR rearrangement—yet still observed rejection of the established male graft [127]. Because no new T‐cell specificities could arise from a RAG‐deficient thymus, this result is difficult to reconcile with the “single non‐self‐antigen” explanation offered.

**TABLE 2:** Peripheral tolerance vs. central adaptation.

	Peripheral tolerance	Peripheral adaptation
Function	Suppression of self‐reactive T cells by Tregs and MDSCs	Tissue repair and remodelling immune responses
Transplant outcomes	Allo‐reactity of T cells toward allo‐antigens	Predominance of homeostatic versus cytotoxic T cell responses Graft adaptation or re‐expression of signal IV for protecting the graft from cytotoxic T cells

The concept of tissue adaptability beyond classical allogenicity is illustrated by skin grafting experiments, in which RAG‐deficient female recipients of syngeneic skin grafts from male mice in the presence of male‐derived self‐reactive T cells accepted grafts from immunocompetent male skin donors but rejected otherwise identical skin grafts from immunodeficient male donors, despite both grafts expressing the same Y‐chromosome antigens [128]. In autografts or isografts, the graft arrives with its own pool of self‐reactive T_RM_ and constitutively expresses signal IV since they have already been activated and established memory in the donor's graft. Surgical injury restimulates the donor's T_RM_ cells and the recipient's immune cells simultaneously, prompting a burst of cytokines and growth factors that accelerate epithelial closure, matrix remodelling, and rapid re‐expression of AdRs in the graft. For example, self‐reactive CD8^+^ T_RM_ secrete amphiregulin (AREG), an EGF‐family growth factor that promotes local tissue remodelling, while their accompanying production of IFN‐γ is accommodated without provoking tissue damage [121]. Because the donor and recipient share already adapted cell–cell interaction memory, this “signal IV” survival circuit is established within hours, allowing postoperative inflammation to be contained and the graft to integrate quickly. In an allograft, however, only the donor T_RM_ population can deliver these tissue‐healing signals, which do not last long in the host and should be replaced by the recipient's T_RM_ cells [129]. When a graft's ECM adapts slowly to its new microenvironment, the resulting tissue injury shifts antigen presentation toward the indirect (cross‐presentation) pathway. This favours cytotoxic T‐cell responses over the homeostatic ones normally elicited by direct recognition, and in turn induces graft injury because AdRs have not yet been fully re‐expressed after surgery. To this end, surgical techniques significantly influence graft outcomes. For example, corneal allografts have been shown to survive when using an X‐shaped incision but are rejected with a circular incision, revealing the impact of surgical methods on graft survival [130]. Additionally, the expression of B7‐H1/PD‐L1 in the cornea and retina illustrates further how tissue‐specific immune regulation can protect grafts from rejection, even in the presence of infiltrating CD4^+^ T cells. Disruption of B7‐H1/PD‐L1 signalling hastens allograft rejection [131], since B7‐H1, through its interaction with PD‐1, transmits survival signals via anti‐apoptotic pathways like Bcl‐xL induction [74, 75, 76, 77, 78]. This mechanism might explain why allografts are rejected following treatments with immune checkpoint inhibitors (ICIs), such as anti‐PD‐1, which block the PD‐L1 survival pathway [132]. Similarly, tumour rejections have been reported following the administration of ICI in patients with melanoma and non‐small cell lung cancer [133]. Interestingly, PD‐1 expressing T cells are not merely exhausted; they actively participate in tissue protection by producing TGF‐β and relaying survival signals to PD‐L1‐positive target cells [134]. Additionally, PD‐1 signalling allows specific regulatory functions to continue as p38 MAPK activity is preserved [135]. Moreover, IFN‐γ‐producing T cells assist in tissue adaptation by upregulating B7‐H1/PD‐L1 on target cells, further establishing a protective immune milieu [136].

The prolonged adaptation period helps explain why allograft acceptance, often termed ‘Woodruff adaptation’, tends to be slower compared to the rapid acceptance seen in autografts or isografts, according to studies by Woodruff and Simpson [137, 138]. The tolerance of allografts in the anterior chamber (AC) of the eye challenges the dominant view that alloreactivity is the sole determinant of graft acceptance or rejection. Despite the perception of the AC as an immune‐privileged site, it has been shown to have lymphatic drainage [139] and to contain lymphocytes and APCs [140, 141]. Notably, studies have demonstrated that allogeneic thyroid tissue transplanted into the AC is tolerated without immune sensitisation but is rejected if the animals were previously sensitised with the same thyroid tissue subcutaneously [137]. This tolerance persists even if the allograft, once adapted within the AC, faces the same immune challenge that previously led to the rejection of a similar subcutaneous graft, suggesting a form of tissue‐specific adaptation [137]. These observations suggest that graft survival hinges on how quickly the graft adapts to the local microenvironment: the AC promotes rapid adaptation, whereas skin delays it. Further supporting this idea, research on NK cell function has shown that NK cells conditioned in an MHC class I‐sufficient environment can eliminate MHC class I‐deficient cells, whereas NK cells developed in an MHC‐deficient environment do not have this capability [142, 143]. This underscores the crucial role of the tissue environment and adaptation in directing immune responses, independently from classical theories of alloreactivity. Although Koene expanded upon the concept of allograft adaptation [144], a comprehensive theoretical framework to understand AdR co‐receptors (AdLs) remained underdeveloped. The adaptation model posits that the adaptability of target tissue primarily, rather than the immune response, dictates the success of organ transplantation. This is underscored by findings that renal graft survival significantly decreases when donors are older than 50 to 55 years, as reported by Terasaki and colleagues [145], indicating that older organs are less adaptable and more prone to cytotoxicity. Conversely, allografts from living‐related donors demonstrate superior survival compared to those from cadavers, likely due to their quicker adaptation to new environments, a phenomenon noted by other groups [146, 147]. Interestingly, certain organs like the liver and kidney are more susceptible to operational tolerance compared to the heart and lungs [148]. This susceptibility is not an intrinsic property of the organs but rather a result of their high regenerative capacity and adaptability [149, 150]. This enhanced adaptability facilitates organ integration with minimal immune suppression, evidenced by studies indicating similar levels of circulating Tregs and suppressive function in both operationally tolerant individuals and healthy subjects [151]. In addition, renal T cells predominantly display an effector/memory phenotype [152], which contributes to graft tolerance by promoting homeostatic signalling [153]. By shifting the focus from solely suppressing the immune response to enhancing tissue adaptation, the adaptation model provides a more holistic understanding of how long‐term graft survival can be achieved. To this end, the organ‐specific differences in T_RM_ longevity, cytokine bias, and niche support help explain why renal transplants typically stabilise faster and exhibit lower rates of chronic remodelling compared with lung allografts. Kidney parenchyma is seeded with long‐lived, MHC‐matched CD8^+^ T_RM_ that are primed to launch a pro‐repair program as soon as ischemia–reperfusion injury (IRI) subsides such that their adoptive transfer to T cell‐deficient mice reduced histological injury after renal IRI [154]. Within hours of injury, renal CD8^+^ T_RM_ cells secrete AREG and low‐dose IFN‐γ. AREG engages EGFR on tubular and endothelial cells, accelerating re‐epithelialisation and limiting apoptosis, while IFN‐γ up‐regulates CXCL9/10, recruiting pro‐resolving macrophages that coordinate matrix turnover. In parallel, tissue‐reactive cytotoxic T cells can eliminate activated fibroblasts, thereby restraining fibrogenesis [65, 155]. Unlike antigenic cross‐presentation, when T cells are primed directly by somatic cells—as we and others have shown in the liver [64, 65, 66, 67, 68]—the dominant outcome is tissue homeostasis. More than 75% of the responding cells secrete trophic mediators that stabilise the tissue, while fewer than 5% adopt a cytotoxic phenotype that targets activated fibroblasts and thus regulates matrix turnover [65]. Consistently, genetic ablation of CD8^+^ T_RM_ markedly worsens epithelial repair and increases fibrosis in murine kidneys, underscoring the efficiency of this resident circuit [156]. Lung T_RM_ cells differ in both phenotype (CD103^hi^, TGF‐β‐imprinted) and lifespan; they are comparatively short‐lived and decline rapidly once antigen is cleared [157]. Like their renal counterparts, however, they are minimally proliferative yet highly functional [158]. These observations suggest a therapeutic angle: transient augmentation of graft‐resident T_RM_—for example with low‐dose IL‐15 complexes [159]—could provide an early wave of reparative cytokines that stabilise the transplanted tissue. The added time would allow the graft itself to remodel its surface proteome, including its antigenic landscape [160].

The adaptation model highlights three synergistic therapeutic approaches. First, administration of growth factors—such as amphiregulin or IGF‐1 analogues—to accelerate ECM remodelling and speed re‐expression of AdRs, shortening the interval before the graft becomes fully integrated with host tissue. Second, administration of IL‐15 or IL‐15/IL‐15Rα complexes to expand and stabilise T_RM_ cells in the graft that supply signal IV survival cues essential for homeostatic repair. Third, minimising peri‐operative injury through ischaemia‐free procurement, normothermic perfusion, and antioxidant conditioning to reduce the burden of dying cells available for cross‐presentation, thereby curbing cytotoxic immunity and allowing tissue‐remodelling responses to predominate.

## Conflicts of Interest

The author declares no conflicts of interest.

## Data Availability

No new data were generated or analysed in the preparation of this review article.
